# Characterization and *In Vivo* Biological Performance of Biosilicate

**DOI:** 10.1155/2013/141427

**Published:** 2013-09-25

**Authors:** Ana Claudia M. Renno, Paulo Sérgio Bossini, Murilo C. Crovace, Ana Candida M. Rodrigues, Edgar Dutra Zanotto, Nivaldo Antonio Parizotto

**Affiliations:** ^1^Department of Biosciences, Federal University of São Paulo, Avenida Ana Costa 95, 11060-001 Santos, SP, Brazil; ^2^Vitreous Materials Laboratory, Department of Materials Engineering, Federal University of São Carlos, Rodovia Washington Luís (SP-310), Km 235, 13565-905 São Carlos, SP, Brazil; ^3^Department of Physiotherapy, Federal University of São Carlos, Rodovia Washington Luís (SP-310), Km 235, 13565-905 São Carlos, SP, Brazil

## Abstract

After an introduction showing the growing interest in glasses and glass-ceramics as biomaterials used for bone healing, we describe a new biomaterial named Biosilicate. Biosilicate is the designation of a group of fully crystallized glass-ceramics of the Na_2_O-CaO-SiO_2_-P_2_O_5_ system. Several *in vitro* tests have shown that Biosilicate is a very active biomaterial and that the HCA layer is formed in less than 24 hours of exposure to “simulated body fluid” (SBF) solution. Also, *in vitro* studies with osteoblastic cells have shown that Biosilicate disks supported significantly larger areas of calcified matrix compared to 45S5 Bioglass, indicating that this bioactive glass-ceramic may promote enhancement of *in vitro* bone-like tissue formation in osteogenic cell cultures. Finally, due to its special characteristics, Biosilicate has also been successfully tested in several *in vivo* studies. These studies revealed that the material is biocompatible, presents excellent bioactive properties, and is effective to stimulate the deposition of newly formed bone in animal models. All these data highlight the huge potential of Biosilicate to be used in bone regeneration applications.

## 1. Introduction

Bioactive materials are a class of materials capable of bonding themselves to living tissues. Chemical bonds are developed through the formation, at the interface between the bioactive material and the living tissue, of a biologically active hydroxycarbonate apatite (HCA) layer, which is chemically and structurally similar to the apatite found in the bone tissue [1].

It has been estimated that 500,000 bone graft procedures are performed annually in the USA and more than 2 million worldwide, promoting a deficit in the availability of allogenic bone grafts. This new reality is stimulating the interest of companies in the supply of bioactive materials for bone regeneration, a market that grows every year [2].

Most of the commercially available products are composed of hydroxyapatite (HA), *β*-tricalcium phosphate (*β*-TCP), or a composite between them [1]. Although there are a great number of options, ceramic materials normally lack osteoinductive properties [1].

**Figure 1:**
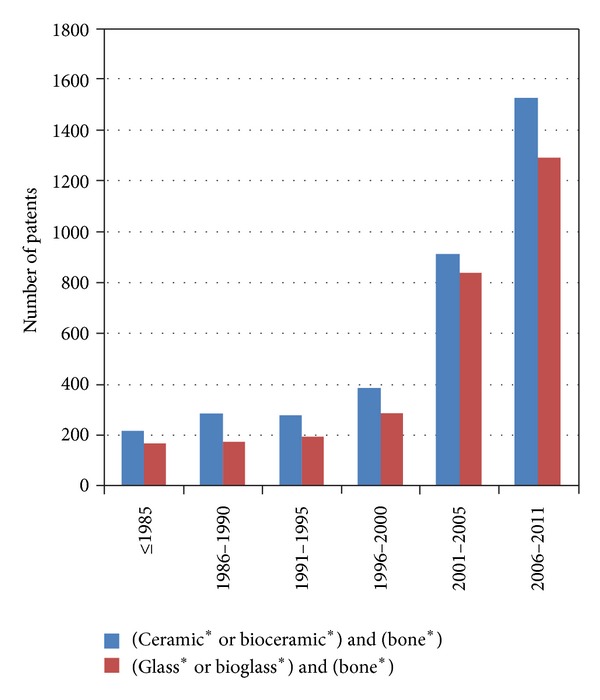
Evolution of the number of patents related to ceramic and glasses used for bone healing.

The growing clinical need is reflected in the number of published papers and deposited patents. A search in Derwent Innovation Index database using the keywords “ceramic*” or “bioceramic*” and “bone*” (topic—January 16, 2012) returned 3,481 hits, whereas a search using the keywords “glass*” or “bioglass*” and “bone*” also returned a great number of hits: 2,950. It is interesting to note that, in both cases, after 2000, there has been a vertiginous increase in the number of patents deposited (Figure 1). Another search in the Web of Science database using the same keywords revealed a similar tendency in the number of papers published in this period.

## 2. Bioactive Glasses and Glass-Ceramics: A Promising Therapeutic Approach

Unlike the common calcium phosphate-based ceramics, bioactive glasses and glass-ceramics show excellent osteoconductive and osteoinductive properties and their degradability rate is high [3]. These materials have been investigated for 4 decades since 45S5 Bioglass was introduced by Hench [4].

Bioactive glasses are able to bond to bone at a significantly higher rate when compared to the bioactive ceramics. The reason for that is related to the rate of formation of the HCA layer on its surface; the faster the formation of the HCA layer is the faster the material will bond to bone [3]. The mechanism of osteoinduction, however, is not fully understood. Nevertheless, research results indicate that, as a bioactive glass degrades, silicon, calcium, and sodium ions and phosphate groups are released in the physiological environment. A combination of these ions is believed to stimulate cells to produce new bone tissue, especially calcium and silicon ions [5]. Molecular biology studies have shown that seven families of genes involved in the osteogenesis process are stimulated by the dissolution products of bioactive glasses [6].

The composition of a bioactive glass determines the reaction kinetics of the glass with the surrounding tissue. When the glass is exposed to body fluids, initially alkali ions are leached and substituted in the glass structure by H^+^ or H_3_O^+^ cations from the fluid (stage I). This causes a local pH increase, causing the rupture of Si–O–Si bonds and the release of silicon in the form of silanol groups (stage II). If the local pH is lower than 9.5, the silanol groups polymerize on the glass surface, forming a silica gel layer (stage III).

**Figure 2:**
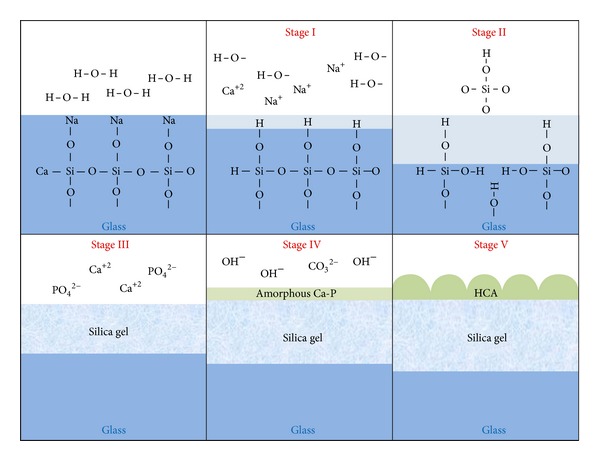
Illustration of the mechanism of hydroxycarbonate apatite (HCA) formation on the surface of a bioactive glass in contact with body fluids.

The open structure of silica gel allows the continuity of ion exchange. Calcium ions and phosphate groups migrate through the silica gel layer, including ions present in the body fluid, and form an amorphous calcium phosphate layer over the silica gel layer (stage IV). After the growth of both silica gel and calcium phosphate layers, the latter incorporates OH^−^ and CO_3_^−2^ groups, giving rise to the crystallization of HCA (stage V) [1]. This sequence of reactions is illustrated in the Figure 2.

**Figure 3:**
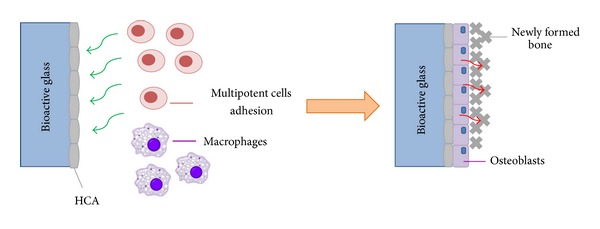
Simplified scheme showing the adhesion of cells to the HCA layer formed in the glass surface (extracted from [8]).

 To form a direct bond to bone, the time for stages IV and V must match the time of natural biomineralization that normally occurs *in vivo*. Although the biological events that precede the bone-bonding process are still being established, it is known that the presence of extracellular proteins, mainly fibronectin, attracts macrophages, mesenchymal stem cells, and osteoprogenitor cells [7]. After that, osteoprogenitor cells proliferate and differentiate in osteoblasts which start the synthesis and deposition of the organic matrix [6]. Thus, the organic matrix undergoes a gradual mineralization process guided by the osteoblastic cells, as simplified in Figure 3. 

However, the greatest disadvantages of bioactive glasses are their low mechanical strength and fracture toughness. In order to overcome these limitations, bioactive glass-ceramics have been developed. Glass-ceramics are materials produced by controlled crystallization of certain glasses. They can be fully crystalline or contain a significant amount of residual glass phase and also be composed of more than one crystalline phase. In general, glass-ceramics show enhanced mechanical properties when compared to the parent glass, as found by Peitl et al. [9] for their glass-ceramics of the Na_2_O-CaO-SiO_2_-P_2_O_5_ system. Another important feature is that glass-ceramic microstructures can be designed to enhance fracture toughness, as in the case of the A/W glass-ceramic, composed of an apatite matrix reinforced by needle-like wollastonite crystals [9, 10]. 

## 3. Characterization of Bone-Bonding Capacity in Bioactive Materials

To analyze the bone-bonding capacity of bioactive materials, *in vitro* tests are commonly used. These tests give information about the kinetics of HCA formation in the surface of the investigated materials. They are preliminary and selective tests performed before the *in vivo* tests, which are expensive and require specialists in laboratory animals, placing of the implants, and sacrifice of the animals for collecting samples. Additionally, *in vitro *tests are more practical and not time consuming. 

The *in vitro* tests consist in the exposure of the material to an acellular solution denominated “simulated body fluid” or SBF. This solution was first developed by Kokubo [10] and simulates the body fluids, containing a quantity and type of ions in the same concentration found in human blood plasma.

**Figure 4:**
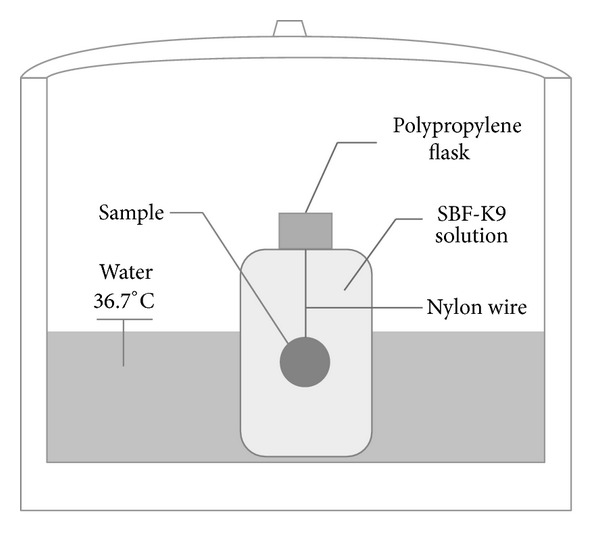
Scheme of the *in vitro* bioactivity test.

To perform *in vitro* tests, the SBF solution is poured in small plastic containers; discs of the testing material are suspended inside the solution by a thin nylon thread (Figure 4). The surface area/volume of the solution ratio is standardized in 0.1 cm^−1^, since a higher value leads to an excessive increase in the reaction kinetics caused by an increase of the pH [10]. The plastic containers are placed in a water bath and kept at 36.7°C (the same temperature of human body) for variable periods of time. After the exposure to the SBF solution, the samples are removed from the plastic containers and dipped in acetone or isopropyl alcohol to stop all reactions. Thus, the surface of these samples is commonly analyzed by Fourier Transform Infrared Spectroscopy (FTIR); this technique is suitable for analyzing chemical changes at the surface of bioactive materials. By this technique it is possible to detect the beginning of the HCA formation. More details about the preparation procedure of the samples and of the SBF solution can be seen in [10]. 

## 4. Biosilicate: A New Glass-Ceramic

**Figure 5:**
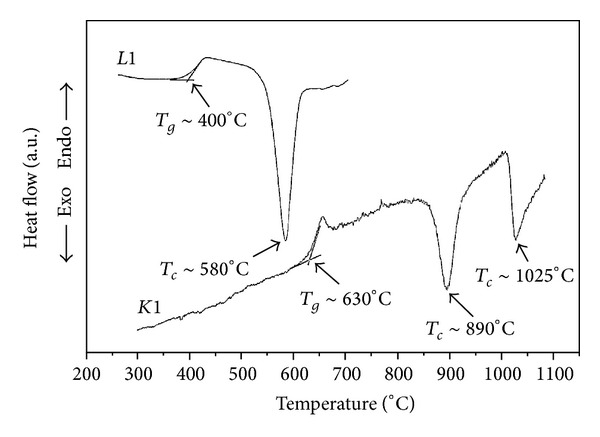
DSC curves of the compositions L1 and K1 [11].

Biosilicate is the designation of a particular composition of a group of fully crystallized glass-ceramics of the Na_2_O-CaO-SiO_2_-P_2_O_5_ system, with additions of Li_2_O and K_2_O.  Table 1 show examples of two compositions named L1 and K1. The patent *WO2004/074199A1 *[11] describes the fabrication procedure of Biosilicate, where glass blocks are obtained by melting reagent grade raw materials (Na_2_CO_3_, CaCO_3_, Na_2_HPO_4_, and SiO_2_) at high temperatures (>1400°C).  Figure 5 shows the DSC curves of L1 and K1 compositions, in which the glass transition temperature, *T*_*g*_, and the temperature of the crystallization peak, *T*_*c*_, are illustrated. To obtain a glass-ceramic, blocks of glass are subjected to heat treatments performed in temperatures between *T*_*g*_ and *T*_*c*_, until full crystallization is reached.

By employing different heat treatments, Biosilicate can be designed to present 1 or 2 crystalline phases, and the microstructure of final glass-ceramic may be controlled by heat treatment. More details regarding the synthesis of this material can be found in [11]. 

**Figure 6:**
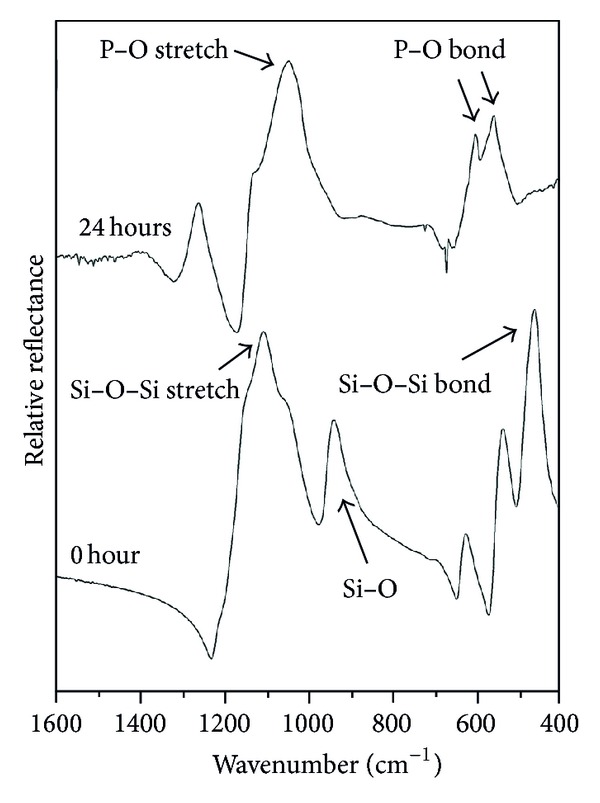
FTIR spectra of Biosilicate nonexposed and exposed to SBF-K9 solution for 24 h, showing the formation of HCA on its surface [11].

Figure 6 shows the FTIR spectra of the nonreacted surface of Biosilicate (0 h of exposure to SBF) and also the FTIR spectra of the surface of a sample which has been in contact with SBF during 24 h. The natural vibrational mode of the nonreacted surface of Biosilicate exhibits peaks at 470 cm^−1^, corresponding to the Si–O–Si bond, and at 1095 cm^−1^, corresponding to the stretch of the Si–O–Si bond. With only 24 h of exposure in SBF (Figure 6) the peaks relative to the Si–O–Si bond already disappeared, giving rise to two sharp peaks at 560 and 602 cm^−1^, both corresponding to the P–O bond. The presence of these peaks in the FTIR spectra indicates the formation of a well-crystallized HCA layer at the surface of Biosilicate.

It is true that, recently, some controversial results about *in vitro* tests using SBF have been mentioned in the literature. In fact, according to Bohner and Lemaitre [12], this test fails in simulating the real and dynamic conditions of a living tissue. According to these authors, several important variables are not taken into account, such as the existence of proteins, the CO_2_ partial pressure in the blood, and the amount of carbonate ions. Moreover, once the SBF solution is supersaturated, heterogeneous nucleation and growth of HCA crystals are sensitive to the presence of contaminants or even scratches inside the plastic container. Nevertheless, we have found excellent reproducibility and also good correlations between *in vitro* tests using SBF and *in vitro* tests using osteoblasts cell culture, as we will show ahead.

The results shown in Figure 6 contradict the idea reported by Li et al. [13] that full crystallization of a bioactive glass can turn it into an inert material. In fact, Peitl et al. [14, 15] have demonstrated that glass crystallization only slightly decreases the kinetics of the HCA layer formation but does not hinder its formation, even in the case of fully crystallized glasses. The result shown in Figure 6 confirms that, despite its high crystallinity, the onset of HCA formation in the surface of Biosilicate is comparable to that of bioglasses, such as the “golden-standard” Bioglass 45S5.

## 5. Biosilicate and Dentin Hypersensitivity

**Figure 7:**
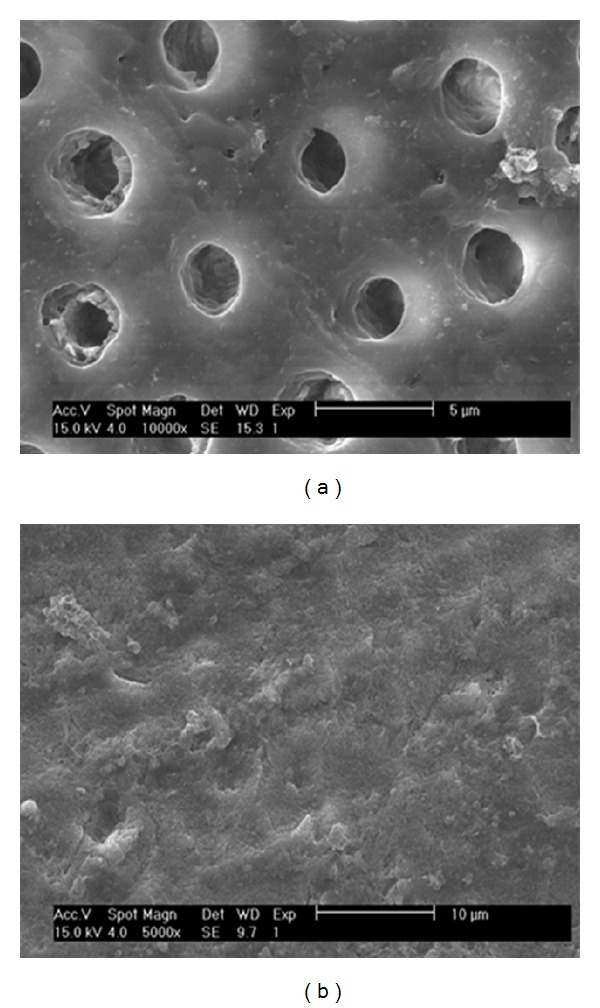
SEM micrographs showing the dentin microchannels before the treatment (a) and after treatment with Biosilicate powder (b) (extracted from [16]).

Biosilicate was first developed in powder form for the treatment of dentin hypersensitivity [16]. When in contact with dentin, Biosilicate particles rapidly react with surrounding tissue inside the dentin microchannels, eliminating the dentinal hypersensitivity by promoting their occlusion (Figure 7).

Tirapelli et al. [16] performed a comparative clinical study to evaluate the Biosilicate (1–20 *μ*m particles) to treat dentine hypersensitivity (DH). The authors showed that Biosilicate is more efficient to treat DH compared to commercially available tooth pastes (Sensodyne and SensiKill), presenting the best clinical performance and providing the fastest treatment to reduce DH pain.

## 6. Biosilicate and Antimicrobial Properties

Furthermore, Martins et al. [17] concluded that the Biosilicate exhibits a wide spectrum of antimicrobial properties, including anaerobic bacteria. The authors assessed the antimicrobial activity of the Biosilicate against anaerobic, microaerophilic, and facultative anaerobic microorganisms. Evaluation of the antimicrobial activity was carried out by three methods, namely, agar diffusion, direct contact, and minimal inhibitory concentration (MIC). In the first 10 minutes of contact between the microorganisms and Biosilicate, there was a drastic reduction in the number of viable cells. Similarly, MIC showed that the Biosilicate inhibited the growth of microorganisms, with variations between ≤2.5 and 20 mg/mL. The lowest MIC values (7.5 to ≤2.5 mg/mL) were obtained for oral microorganisms.

## 7. Biosilicate and Bone Repair

Many studies have been showing the stimulatory effects of Biosilicate on bone metabolism and on the acceleration of fracture consolidation [18–20]. Comparing the growth of osteogenic cells on Biosilicate and Bioglass 45S5 disks for a period of up to 17 days, Moura et al. [21] found that, although no significant differences were detected in terms of protein content and alkaline phosphatase activity at days 11 and 17, Biosilicate supported significantly larger areas of calcified matrix at day 17. Results indicate that full crystallization of bioactive glasses in a range of compositions of the system P_2_O_5_-Na_2_O-CaO-SiO_2_ may promote enhancement of *in vitro* bone-like tissue formation in an osteogenic cell culture system. Moreover, Renno et al. [22], in *an in vitro* study, observed that osteoblastic cells were successfully grown on discs composed of a glass-ceramic composite. 

In view of the aforementioned, it was hypothesized that Biosilicate was effective in accelerating bone healing and could provide a bone graft with additional advantages for clinical use. Thus, our group developed a sequence of tests, investigating the effects of Biosilicate, with 2 different particle size distributions (180–212 *μ*m and 300–355 *μ*m) on bone healing in a tibial bone defect model in rats. It was observed that, 20 days after the injury, bone defects filled with particles of Biosilicate (180–212 *μ*m in diameter) showed improved biomechanical properties and a higher amount of newly formed bone in the area of the callus compared to control animals and to animals treated with 45S5 Bioglass [23, 24].

Moreover, Roriz et al. [25] investigated the efficacy of Biosilicate and a bioactive glass (Biogran) placed in dental sockets in the maintenance of alveolar ridge and in the osseointegration of Ti implants in dogs. Twelve weeks after implantation, samples were processed, and the authors demonstrated that the presence of Biosilicate or Biogran particles preserved alveolar ridge height without affecting its width. However, no significant differences in terms of bone-implant contact and mineralized bone area between threads were detected among Biosilicate, Biogran, and the nonimplanted group.

Azenha et al. [26] investigated the histological and histomorphometric bone responses induced by Biosilicate and 45S5 Bioglass implants in a femoral bone defect model. Eight and 12 weeks after surgery, histological examination did not reveal persistent inflammation or foreign body reaction at implantation sites. The area of bone formation at the cortical portion in the animals treated with Biosilicate was significantly higher compared to those treated with 45S5 Bioglass. 

Furthermore, the effects of the association of Biosilicate and low level laser therapy (LLLT) on bone healing in rats was investigated. Oliveira et al. [27] used 40 male Wistar rats divided into 4 groups: bone defect control group (CG), bone defect filled with Biosilicate group (BG), and bone defect filled with Biosilicate and irradiated with LLLT at 120 J cm(−2) group (BG 120). The size of particle used for Biosilicate was 180–212 micrometers. Laser irradiation was initiated immediately after the surgery procedure, and it was performed every 48 h for 14 days. Fourteen days after surgery, the three-point bending test revealed that the structural stiffness of the groups CG and BG was higher than the values of the groups BG120. Morphometric analysis revealed no differences between the control group and the Biosilicate group. Interestingly, the groups treated with Biosilicate and laser (BG 60 and BG120) showed statistically significant lower values of newly formed bone in the area of the defect when compared to CG and BG. The findings suggest that although Biosilicate exerts some osteogenic activity during bone repair, laser therapy is not able to modulate this process.

Interestingly, different results were found in osteopenic rats. Bossini et al. [28] investigated the effects of LLLT and Biosilicate (180–212 *μ*m) on bone consolidation in osteopenic rats. The histopathological analysis showed that bone defects were predominantly filled with the biomaterial in specimens treated with Biosilicate. Moreover, laser therapy was able to increase collagen, Runx-2, VEGF, and COX-2 expression in the circumjacent cells of the biomaterial. Moreover, the morphometric analysis revealed that the Biosilicate plus laser groups showed a higher amount of newly formed bone. 

Furthermore, the good performance reached by Biosilicate during *in vitro* and *in vivo* tests encouraged studies that could lead to the development of scaffolds from Biosilicate, which could constitute a promising alternative for the treatment of bone injuries. 

## 8. Conclusion

The present review demonstrates that Biosilicate is biocompatible, presents both osteogenic and angiogenic potential, and can accelerate the bone healing process in animal models. These data highlight the huge potential of Biosilicate to be used as a bone graft. But further *in vivo* long-term studies should be carried out to provide additional information concerning the mechanisms involved in the stimulation of bone by this new, highly bioactive material before Biosilicate can be used with confidence as a treatment within the clinical setting.

## Figures and Tables

**Table 1:** Chemical composition of Biosilicates L1 and K1 (wt%) [11].

Composition (wt%)	Li_2_O	Na_2_O	K_2_O	CaO	SiO_2_	P_2_O_5_
L1	7.4	22.0	—	22.0	44.9	3.7
K1	—	—	23.75	23.75	48.5	4.0
